# The early‐life environment and individual plasticity in life‐history traits

**DOI:** 10.1002/ece3.4749

**Published:** 2018-12-30

**Authors:** Ornela De Gasperin, Ana Duarte, Sinead English, Alfredo Attisano, Rebecca M. Kilner

**Affiliations:** ^1^ Department of Zoology University of Cambridge Cambridge UK; ^2^ Department of Ecology and Evolution University of Lausanne Lausanne Switzerland; ^3^ Science and Engineering Research Support Facility (SERSF) University of Exeter Penryn UK; ^4^ School of Biological Sciences University of Bristol Bristol UK; ^5^ Museum and Institute of Zoology Polish Academy of Sciences Warsaw Poland

**Keywords:** burying beetles, developmental plasticity, early‐life effects, environment matching, informational model, life‐history trade‐offs, phoretic mites, silver spoon, somatic model

## Abstract

We tested whether the early‐life environment can influence the extent of individual plasticity in a life‐history trait. We asked: can the early‐life environment explain why, in response to the same adult environmental cue, some individuals invest more than others in current reproduction? Moreover, can it additionally explain why investment in current reproduction trades off against survival in some individuals, but is positively correlated with survival in others? We addressed these questions using the burying beetle*, *which breeds on small carcasses and sometimes carries phoretic mites. These mites breed alongside the beetle, on the same resource, and are a key component of the beetle's early‐life environment. We exposed female beetles to mites twice during their lives: during their development as larvae and again as adults during their first reproductive event. We measured investment in current reproduction by quantifying average larval mass and recorded the female's life span after breeding to quantify survival. We found no effect of either developing or breeding alongside mites on female reproductive investment, nor on her life span, nor did developing alongside mites influence her size. In post hoc analyses, where we considered the effect of mite number (rather than their mere presence/absence) during the female's adult breeding event, we found that females invested more in current reproduction when exposed to greater mite densities during reproduction, but only if they had been exposed to mites during development as well. Otherwise, they invested less in larvae at greater mite densities. Furthermore, females that had developed with mites exhibited a trade‐off between investment in current reproduction and future survival, whereas these traits were positively correlated in females that had developed without mites. The early‐life environment thus generates individual variation in life‐history plasticity. We discuss whether this is because mites influence the resources available to developing young or serve as important environmental cues.

## 1 INTRODUCTION

**Figure 1:**
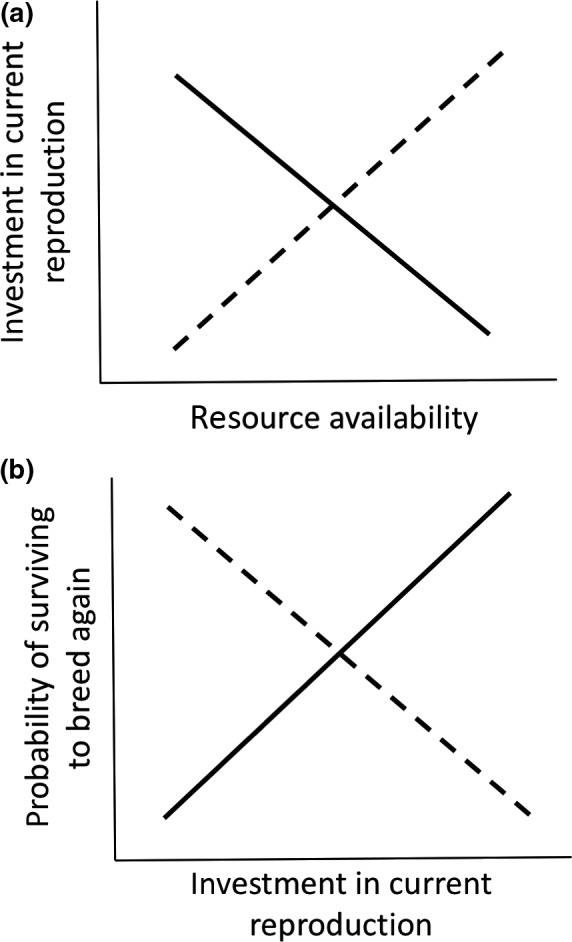
Illustration of the two types of individual variation in plasticity that we seek to explain. (a) Variation in the extent of investment in current reproduction in relation to resource availability. Why do some individuals reduce their reproductive investment when in a poor environment (dashed black line) whereas other individuals increase their investment (solid black line). (b) Relationship between investment in current reproduction and probability of surviving to breed again. Why is there a positive correlation in some individuals (solid black line) but a negative correlation in others (dashed black line)?

Phenotypic plasticity is classically defined as variation in the phenotype that is induced when a single genotype is exposed to different environments (Pigliucci, 2001; West‐Eberhard, 2003). Although it is well established that the expression of diverse traits can be environmentally induced (Bennett & Murray, 2014; Charmantier et al., 2008; Kuzawa, McDade, Adair, & Lee, 2010; Nussey, Clutton‐Brock, Elston, Albon, & Kruuk, 2005), it is not clear why individuals vary in the extent of trait change upon exposure to the same environmental cue (Figure 1a). Some of this variation can be due to genetic variation in the slope of the reaction norm (e.g., Scheiner, 1993; Scheiner & Lyman, 1989). Furthermore, recent theoretical work has considered whether environmental conditions experienced in early life might account for some of the individual variation in the extent of plasticity shown in adult life (Dingemanse & Wolf, 2013; Nettle & Bateson, 2015; Stamps & Frankenhuis, 2016).

### 1.1 Plasticity in current reproductive investment: two types of individual variation

Here, we investigate whether conditions in early life can explain individual variation in the plasticity of a key life‐history trait: current reproductive investment. We focus on two different aspects of plasticity connected with this trait (illustrated in Figure 1). First, we are interested in explaining individual variation in the slope (and elevation) of the reaction norm relating absolute levels of investment in current reproduction to current environmental conditions (Stearns, 1992; Nussey, Wilson, & Brommer, 2007, Figure 1a). Figure 1a illustrates extreme individual variation in the slope of such a reaction norm. Previous work suggests that the early‐life environment could account for some of this variation (e.g., Lindström, 1999; Lummaa & Clutton‐Brock, 2002; Monaghan, 2008; Cartwright, Nicoll, Jones, Tatayah, & Norris, 2014).

The second type of plasticity concerns the relationship between current reproductive investment and probability of surviving to breed again (Figure 1b). Although these two life‐history traits are commonly related to each other (Stearns, 1989, 1992), the direction of this relationship can vary from individual to individual (Reznick, Nunney, & Tessier, 2000). In some individuals, increased investment in current reproduction causes an allocation of resources away from investment in survival, yielding a negative relationship. Other individuals, however, can invest substantially in both current reproduction and survival (Reznick et al., 2000), yielding a positive relationship between the two. Again, early‐life conditions might explain the sign of the relationship between current reproductive investment and survival.

### 1.2 The role of the early‐life environment in explaining individual variation in plasticity

How, exactly, might the early‐life environment cause variation in the plasticity of current reproductive investment, and the direction of its relationship to survival? Two possibilities have been identified by recent theoretical models, and they are not mutually exclusive. Somatic models assume that the early‐life environment functions to provide key resources to the developing individual (DeWitt, Sih, & Wilson, 1998; Monaghan, 2008). This can account for both types of variation illustrated in Figure 1. For example, the more resources an individual acquires during its development, the greater its capacity to mobilize the resources required for phenotypic plasticity in later life (Bennett & Murray, 2014; Snell‐Rood et al., 2015). Furthermore, with greater resources at its disposal, an individual can invest in both current reproduction and in surviving to breed again (the silver spoon effect), yielding a positive relationship between current reproductive investment and survival (Reznick et al., 2000). Only when resources are more limited during development will increased investment in current reproduction cause a trade‐off in adult life and result in a reduced probability of surviving to breed again (Reznick et al., 2000; Snell‐Rood et al., 2015).

Informational models differ from somatic models by assuming that the early‐life environment functions to provide information that can guide phenotypic changes (Frankenhuis & Panchanathan, 2011; Nettle & Bateson, 2015; Stamps & Frankenhuis, 2016). If individuals sample their environment at intervals, they can use Bayesian updating to gain a more accurate and complete assessment of environmental conditions before committing to a particular phenotype (English, Fawcett, Higginson, Trimmer, & Uller, 2016; Frankenhuis & Panchanathan, 2011; Stamps & Frankenhuis, 2016). Individuals that receive a more consistent set of cues can strategically commit to a phenotype sooner than might be expected in the absence of such cues (Frankenhuis & Panchanathan, 2011). The variation seen in adult life (illustrated in Figure 1a) can thus be explained by the cues perceived during development. If these cues match the cues perceived in adulthood, for example, it might yield increased investment in current reproduction—because an individual has greater certainty that environmental conditions will yield high fitness returns on greater investment in current reproduction (English et al., 2016; Frankenhuis & Panchanathan, 2011; Stamps & Frankenhuis, 2016). By contrast, if the cues perceived during development differ from those perceived in adult life, it might yield reduced investment, owing to greater levels of uncertainty about true environmental conditions (English et al., 2016; Frankenhuis & Panchanathan, 2011; Stamps & Frankenhuis, 2016). Likewise, accurate information about environmental quality might be used strategically to determine whether the relationship between current reproductive investment and survival is positive or negative (Reznick et al., 2000). If individuals receive matching cues that the chance of successful future reproduction is very low, for example, then they might strategically reallocate resources away from future reproduction toward current reproduction (e.g., Cotter, Ward, & Kilner, 2011). This would yield a negative relationship between current reproductive investment and the probability of surviving to breed again. By contrast, mismatching cues provide less certainty. The default strategy could then be for individual quality to determine the extent of investment in current reproduction and the probability of surviving to breed again, yielding a positive relationship overall (Reznick et al., 2000).

### 1.3 The study system: burying beetle *Nicrophorus vespilloides*

Here, we describe experiments on the burying beetle, *Nicrophorus vespilloides*, that test these ideas. Reproduction by burying beetles centers on the carcass of a small vertebrate, which the beetles roll up and bury below ground (Pukowski, 1933; Scott, 1998). The larvae hatch from eggs laid in the soil and crawl to the carcass, which becomes an edible nest where they are tended by their parents (Pukowski, 1933; Scott, 1998). The larval stage ends when larvae cease feeding and crawl away into the soil to pupate.

Burying beetles exhibit considerable variation in the plasticity of their life‐history traits (Cotter et al., 2011; Pilakouta, Halford, Rácz, & Smiseth, 2016; Ward, Cotter, & Kilner, 2009). For example, females that experience competition with other burying beetles over carcasses increase their expenditure on their first brood and reduce their survival (Creighton, Heflin, & Belk, 2009; Pilakouta et al., 2016). If there is intense competition for a carcass, then, it is unlikely that a female will be able to secure a second carcass and breed again. However, females vary in the extent to which they reallocate resources to current reproduction in response to competition (Pilakouta et al., 2016). Mechanistically, this can be achieved if females eat less of the carcass themselves and allow their brood to eat more (Boncoraglio & Kilner, 2012; Creighton et al., 2009). We investigate whether patterns of resource allocation can be explained by variation in the early‐life environment.

We focus on one element of the early‐life environment in particular: phoretic mites, of the *Nicrophorus*‐specific *Poecilochirus carabi* species complex, which breeds alongside the female on the carcass (Schwarz, Starrach, & Koulianos, 1998). These mites are relatively large, are easily seen while they are on the beetle, and are carried by both sexes. Mites travel on adult beetles as deutonymphs, the stage in their life cycle specialized for transportation (Schwarz & Müller, 1992). Deutonymphs alight on the carcass, molt to become adults, mate, lay their eggs, and then die. Newly hatched mites are already present on the carcass, walking, and potentially feeding on the flesh, when the burying beetle larvae hatch and crawl through the soil to take up residence on the carrion nest. During reproduction, adult beetles and larvae frequently encounter mites because each species moves extensively over the carrion nest. This suggests that beetles are able to detect the density of mites on the carcass (although we do not know which cues the beetles use to assess whether mites are on the carcass). The new generation of mites stays on the carrion until the parent beetles depart: c.90% of deutonymphs climb on to the parents to disperse, rather than dispersing on the larvae (Schwarz & Müller, 1992). Therefore, in nature, an individual can be exposed to mites during development and not carry mites in adult life (or vice versa).

Mites are thus a key part of the beetle's developmental environment. Furthermore, mites can potentially function in the ways proposed by both the somatic model and the informational models described above. In keeping with the somatic model, mites could limit the resources available on the carrion to developing larvae because they are rivals for resources: the more resources that are consumed by mites, the less there is left for nourishing the larvae (De Gasperin & Kilner, 2016). In addition, mites potentially provide an environmental cue for beetles, in keeping with the informational models. Mite reproduction is tied to beetle reproduction: the more beetles there are breeding in a population, the greater the number of mites there are overall. Mites also move horizontally between adult burying beetles, whenever *Nicrophorus* species congregate to feed or mate opportunistically. We thus expect mite numbers to swell with beetle population density during the breeding season and also to be approximately evenly distributed among adults through horizontal transfers. This means that mite density could act as a cue for beetle population density and therefore the likely extent of competition for a carcass—although this possibility has not yet been tested. Furthermore, and again in keeping with the informational models, individual beetles can be exposed to mites as larvae during development and again as adults when they breed. This means individuals can repeatedly sample this environmental cue before deciding how to invest in current reproduction.

We carried out a laboratory experiment with a 2 × 2 fully factorial design where we manipulated whether or not female beetles were exposed to mites at two different life stages: during development as a larva and when they reproduced for the first time. In all four treatments, we measured a female's investment in current reproduction by quantifying the average mass of her larvae at dispersal, and we measured her survival to future reproduction by quantifying life span after reproduction. With this design, we determined whether exposure to mites during early life and exposure to mites during first reproduction, each independently influenced: (a) the extent of investment in current reproduction and (b) the direction of the relationship between current reproductive investment and survival after reproduction. Using field observations, we also tested whether the informational models could apply to burying beetles and their mites, by assessing whether (c) mite density per beetle provides a reliable cue of burying beetle population density.

## 2 MATERIALS AND METHODS

### 2.1 Laboratory experiment

All the beetles used in this experiment came from a stock population. The establishment and maintenance of this population are described elsewhere (De Gasperin & Kilner, 2016; De Gasperin, Duarte, & Kilner, 2015). Mites were harvested from freshly caught beetles and bred separately from the stock population using methods described elsewhere (De Gasperin & Kilner, 2016; De Gasperin et al., 2015). For logistical reasons, this experiment was staged over two successive blocks, focused only on females, and only used mites originating from Byron's Pool (see field data collection below).

### 2.2 Step 1: developing with or without mites

**Figure 2:**
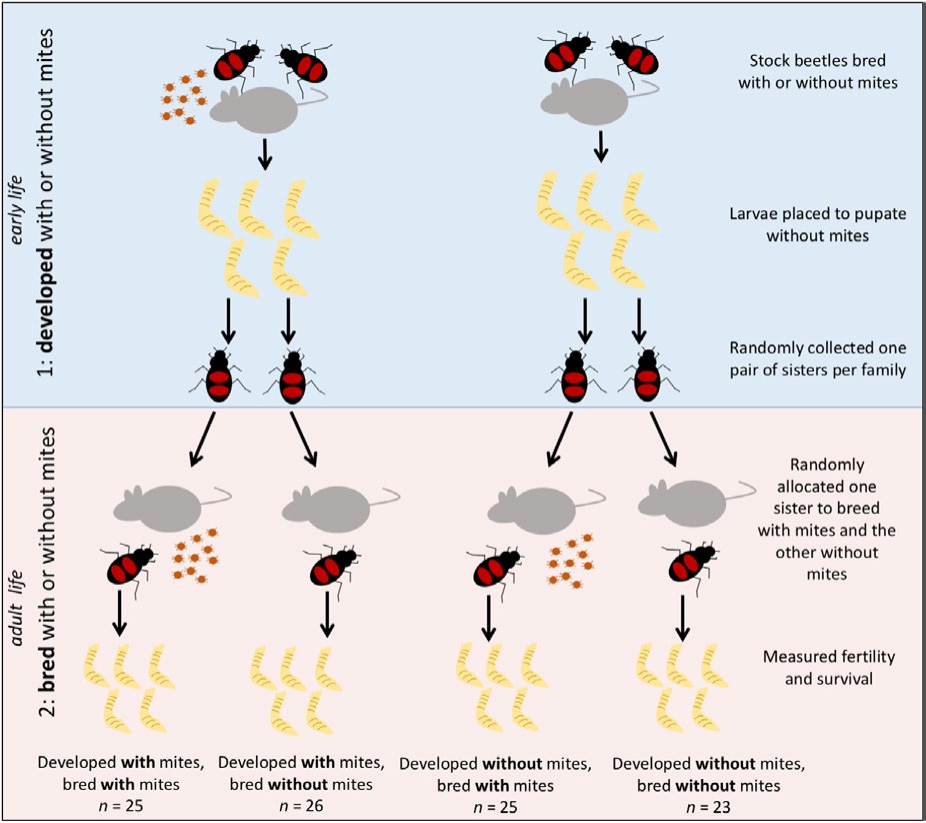
The design of the experiment. Females were exposed to mites twice during their lives: during their development on the carcass as larvae (“early life”) and during their first reproductive event (“adult life”). Mites were placed on the soil surrounding the carcass in both breeding events, and once each breeding event was finished (8 days after pairing the adults), mites were removed from the larvae (early‐life stage) and from the adults (adult life stage)

We manipulated a female's exposure to mites in two steps: (a) as a larva and (b) as an adult, and measured the effect of such exposure on her investment in the first brood and on her subsequent survival (see Figure 2). Step 1 also formed a separate, self‐contained experiment in its own right, which is published elsewhere (De Gasperin et al., 2015). For this step, we set up pairs of sexually mature, virgin beetles to breed on an 8–15 g carcass inside its own plastic container (28.5 × 13.5 × 12 cm), which was divided into two by a cardboard partition containing a one‐way valve. The valve allowed beetles to leave the breeding chamber, but not to return (see figure 1 in De Gasperin et al., 2015). We used boxes that mimicked natural conditions to allow parents to carry mites away from the breeding event as they would in nature, thus avoiding unnatural costs of developing alongside mites when parents cannot leave the nest (De Gasperin & Kilner, 2016; De Gasperin et al., 2015).

Pairs were cast into two treatments: In one, they received 10 mites when pairs were set up (wild‐caught *N. vespilloides *pairs carry on average 8–16 mites as they arrive at a carcass to breed; Schwarz & Müller, 1992); in the other, they had no mites. Mites were added to the breeding box, on the soil, when beetles were paired and moved quickly onto the carcass. Eight days after pairing the adults, when the larvae are fully developed and there are no traces of the carrion left, we collected the dispersing larvae and weighed the brood. We ensured that no dispersing larvae carried mites. Therefore, any exposure to mites experienced by the larvae in this experiment was confined to the period during development on the carcass (8 days). The mass and size of these broods did not differ according to the mite treatment (see De Gasperin et al., 2015). We placed each brood in its own “eclosion box” (12 × 8 × 2 cm), subdivided into 25 1 × 1 cm cells with one larva per cell. We then filled each box with soil and sprayed it with water once to maintain humidity, and closed each box. The larvae remained in these boxes until pupation was complete (~3 weeks), at which point we randomly collected one pair of sisters from each brood. Each of these adult females was kept alone in a small, individual plastic container filled with moist soil (12 × 8 × 2 cm), and fed twice a week with minced beef until they reached sexual maturity, when they were used in Step 2 of the experiment.

### 2.3 Step 2: reproducing with or without mites

Each sister was randomly allocated to one of two treatments: either breeding in the presence or absence of mites. Mites were added to the breeding box using the procedure described in Step 1. Thus, we had four experimental treatments: raised with mites, bred without mites (*n* = 26 successful replicates); raised without mites, bred without mites (*n* = 23 successful replicates); raised with mites, bred with mites (*n* = 25 successful replicates); and raised without mites, bred with mites (*n* = 25 successful replicates).

At 15–20 days post‐eclosion, females were paired with a virgin, sexually mature, unrelated stock male (that developed without mites). Each pair was placed inside a plastic container (17 × 12 × 6 cm) filled with ~3 cm of moist soil and was given an 8–15 g carcass (mean = 10.85; *SD* = 1.63). At this point, 10 mites were introduced, as described previously, into the boxes of those pairs assigned to breed alongside mites. All males were removed in the afternoon before hatching (~56 hr after pairing) to eliminate any confounding effects due to post‐hatching paternal care.

Eight days after pairing, as larvae were dispersing from the carcass, we counted them, weighed the brood, and calculated the average larval mass (by dividing total brood mass by brood size). At this point, we anaesthetized all females using CO_2_, and removed and counted all the second‐generation mites dispersing on them. We also anaesthetized the females that bred without mites, and simulated the removal of mites from them. Adult females were then kept individually in plastic boxes filled with moist soil (they were not in contact with mites again). We also measured their pronotum width, a standard technique for measuring beetle size (Otronen, 1988). We fed them twice a week until they died. Thus, we measured their fecundity the first time they bred, and their subsequent life span, as a function of their two exposures to mites. The opportunistic nature of the burying beetle's reproduction means life span is a good proxy for residual fitness, as has been explained and justified in detail before (De Gasperin & Kilner, 2015; Kilner et al., 2015). The shorter a beetle's life, the less likely it is that it will survive to find another carcass for reproduction.

### 2.4 Field data collection

To understand whether burying beetles could use mites as a cue for population density, we collected field data on the abundance of *Nicrophorus *beetles in general, including *N. vespilloides* specifically*,* and their mites. We sampled wild *Nicrophorus *spp. beetles from spring to autumn of 2013 at two field sites, Byron's Pool (BP) (52°10ʹ5ʺN, 0°7ʹ55ʺE) and Wicken Fen (WF) (52°31ʹ06ʺN, 0°29ʹ13ʺE), each in Cambridgeshire, UK. Note that it is highly unlikely that individual beetles could travel between these sites (Pascoal & Kilner, 2017). Therefore, putative cues from mites need only predict the local environment at each site, rather than the environment across both sites, to be of strategic value to a beetle. At each site, beetles were collected using Japanese beetle traps (BP: 6 traps; WF: 12 traps) filled with soil and baited with a mouse carcass (~15 g), set along a transect at intervals of ~120 m (BP: 1 transect; WF: 2 transects placed 1 km from each other). Every fortnight, we tipped the contents of each trap into its own plastic box (17 × 12 × 6 cm) and transported them to our laboratory. Here, we collected individuals from each box, identified species and sex, and anesthetized each individual with CO_2_ to remove the mite load. We removed the mites attached to each beetle with tweezers and with a fine paintbrush and counted them.

### 2.5 Statistical analysis: laboratory experiment

We conducted all the statistical analyses in R (R Core Team, 2014) (v. 3.3.0). To analyze the female's investment in current reproduction, we used two general linear mixed effects models (lmer function, lme4 package, Bates, Maechler, Bolker, & Walker, 2015), one analyzing variation in brood size and the other analyzing variation in average larval mass (obtained by dividing the total brood mass by the brood size). Because, brood size and brood mass are highly correlated so we just analyzed brood size. As recommended by Zuur, Ieno, Walker, Saveliev, and Smith (2009a, 2009b), we first compared the full models including the sisters’ family of origin as a random effect nested within the experimental block, and full models including the block as a fixed effect. After comparing the models, we kept the experimental block as a fixed effect, and only left the females’ family of origin as a random effect. In every model, we included as explanatory variables the developmental environment experienced by each female (with or without mites), her environment when she reproduced (with or without mites) and the interaction between these variables. We also included as covariates the size of the female and the mass of the carcass she bred upon as an adult to control for these potential confounding variables (Boncoraglio & Kilner, 2012; De Gasperin & Kilner, 2016). Finally, we included the experimental block as a covariate with two levels, block 1 and 2. When we used average larval mass as the response variable, we also included the size of the brood as a covariate. In this model, we found heteroscedasticity in the residuals, as a function of brood size and of the carcass mass. Hence, we fitted a generalized least squares model using the combined variance structure (*varComb*), allowing for the average larval mass to have a *varPower* variance structure as a function of the carcass mass, and a *varExp* variance structure as a function of the size of the brood, to correct for this (Zuur et al., 2009a, 2009b). Furthermore, when we used average larval mass as the response variable, we also found an outlier, a female who produced larvae of 0.0637 g (when the median average larval mass produced by the females was around 0.14 g). We removed this outlier and repeated the analysis (the results were not influenced by it, yet all results presented analyzing variation in the average larval mass have excluded this outlier). For this model, we examined the normalized residuals to assess the validity of this model (Zuur et al., 2009a, 2009b). In the model analyzing variation in brood size (general linear mixed model), we examined the residuals to validate the model. To analyze female life span, we used a Cox‐proportional hazards model with mixed effects (coxme package in R; Therneau, 2015). Again, we included as explanatory variables the developmental environment experienced by each female (with or without mites), her environment when she reproduced (with or without mites) and the interaction between these variables. We also included the size of the female, the mass of the carcass she bred upon as an adult, and the experimental block as covariates. We included the female's family to control for having sisters across adult treatments. We obtained effect sizes, associated standard errors, and *p*‐values using the “summary” function, and using the “ANOVA” function, with type III sum of squares for models with interactions, and with type II sum of squares for models without interactions (Fox et al., 2012). For all analyses, we present full models as recommended by Forstmeier and Schielzeth (2011). Note that for all subsequent analyses we followed the same procedure.

While carrying out these experiments, we observed that the number of second‐generation mites on a carcass at the end of reproduction varied by an order of magnitude within mite treatments (from 20 to 300), even though we had added the same number of mites at the start when beetles were paired. We attribute this to chance variation in the sex ratio of the 10 deutonymphs added at the start of the breeding event (Nehring & Müller, 2009). It is not possible to sex deutonymphs without destroying them. Therefore, in a set of post hoc analyses, we investigated whether the number of mites present at the end of the female's breeding event explained variation in the traits we measured (brood size, average larval mass, female life span). Note that these analyses were not part of our a priori predictions. These analyses focused only on the subset of females that had mites during reproduction (because otherwise by including females from the no mite reproductive environment, the distribution of the data would be skewed by a large number of 0 values, making the results of any statistical analyses difficult to interpret). Roughly half of these females that bred alongside mites also experienced mites during development: The rest did not.

We used the same approach for analyzing the data as described above. This time we included as explanatory variables the number of second‐generation mites dispersing on the female (log‐transformed), the developmental environment experienced by the female (with or without mites), and the interaction between these variables. Previous analyses in our laboratory show that the number of second‐generation mites dispersing on the adults strongly correlate with the total number of mites at the end of the breeding event (Duarte, unpub data, Pearson's *r = *0.78; *t* = 3.82; *df* = 9, *p* = 0.0040). Thus, to analyze variation in the size of the brood, we used a general linear model (note that we no longer had sisters repeated between treatments), and included as explanatory variables the developmental environment experienced by each female (with or without mites), the number of mites present at the end of the breeding event (log‐transformed number of second‐generation mites dispersing on the female), and the interaction between these variables. We also included as covariates the size of the female and the mass of the carcass she bred upon as an adult, and the experimental block. When we analyzed the average larval mass, again we found heteroscedasticity in the residuals, as a function of the experimental block. In particular, there was more variance in the size of the larvae in the first than in the second experimental block. Hence, we fitted a generalized least squares model using the *varIndent* variance structure as a function of the experimental block, to correct for this (Zuur et al., 2009a, 2009b). Afterward, we examined the normalized residuals to assess the validity of this model (Zuur et al., 2009a, 2009b). To examine the relationship between current reproductive investment and survival, we used female life span as the response variable in a Cox‐proportional hazards model (survival package; Therneau & Lumley, 2015). We included as explanatory variables the average larval mass that she produced in her first breeding event, whether she developed with or without mites, and the interaction between these variables. We also included as explanatory variables the mass of the carcass, the size of the female, and the experimental block as covariates. To avoid large notations in the hazards ratios associated with the units of the average larval mass, we standardized this variable (note that the results were the same if we had included the average larval mass without standardizing). We assessed the proportional hazards assumption for each covariate included in the survival models using the cox.zph function, from the “survminer” package (Kassambara, Kosinski, & Biecek, 2017).

We also tested whether mites directly influence female survival, by using again female life span as the response variable in a Cox‐proportional hazards model (survival package; Therneau & Lumley, 2015), and including as explanatory variables the final number of second‐generation mites dispersing on the female (log‐transformed), whether she developed with or without mites, and the interaction between these variables. We also included as explanatory variables the mass of the carcass, the size of the female, and the experimental block as covariates. We assessed the proportional hazards assumption for each covariate included in the survival models using the cox.zph function, from the “survminer” package (Kassambara et al., 2017).

We ran further analyses to address whether any effect of mites on life‐history trade‐offs could be due to direct effects on female condition. We tested whether the size of the adult female was related to the presence of mites during her development, using a general linear mixed model, with female size as a response variable and the presence or absence of mites during her development as an explanatory variable. We included the experimental block as a covariate.

### 2.6 Statistical analysis: field data

We investigated the relationship between number of beetles in natural populations and the number of mites carried per beetle. Specifically, we analyzed the relationship between the average number of mites per *Nicrophorus* spp beetle and the number of *Nicrophorus* spp beetle individuals. We calculated the average number of mites per *Nicrophorus* spp beetle as the sum of all the mites found on every *Nicrophorus* spp. beetle at one site in one field collection day, divided by the total number of *Nicrophorus* spp. beetles collected at that site in that field collection day. Because both the average number of mites per *Nicrophorus *beetle and the total number of beetles collected per day per site were not normally distributed (Shapiro test: *W* = 0.75; *p* > 0.0001 and *W* = 0.87; *p* = 0.0045, respectively), we log‐transformed these variables. We ran a linear model, in which the response variable was the average number of mites per beetle per site, and explanatory variables were the total number of beetles collected per day per site (log‐transformed), the site, and the interaction between these two variables.

## 3 RESULTS

### 3.1 Can individual variation in the extent of current reproductive investment and in survival be explained by exposure to mites during a female's own development?

We found that the interaction between the presence and absence of mites during development and reproduction did not influence brood size, average larval mass, or female life span (Table 1). Furthermore, neither the presence or absence of mites during development (generalized least squares model, after removing the interaction effect: value = 0.004; *SE* = 0.003; *t = *1.07; *p = *0.28) nor during reproduction (generalized least squares model, after removing the interaction effect: value = −0.002; *SE* = 0.003; *t *= −0.79; *p = *0.42) explained variation in the average larval mass. Similarly, neither the presence or absence of mites during development (general linear mixed model, after removing the interaction effect: *χ* ^2^ = 0.001; *p = *0.96) nor during reproduction (general linear mixed model, after removing the interaction effect: *χ* ^2^ = 0.45; *p = *0.50) explained variation in the size of the brood.

**Table 1:** Effect of the mite treatments on female life‐history traits; *n* = 25 developing without mites and breeding with mites; *n* = 25 developing with mites and breeding with mites; *n* = 26 developing with mites and breeding without mites and *n* = 23 developing without mites and breeding without mites

Explanatory variables	*χ* ^2^	*t*	*p*
*Brood size (linear mixed effects model)*			
Intercept	3.19	1	0.07
Development (with or without mites)	0.31	1	0.57
Reproduction (with or without mites)	1.08	1	0.29
Carcass mass	2.27	1	0.13
Female size	0.03	1	0.85
Experimental block	0.78	1	0.37
Development (with or without mites) * Reproduction (with or without mites)	0.63	1	0.42

Explanatory variables	Value	*SE*	*t*	*p*
*Average larval mass (generalized least squares with mixed effects)*				
Intercept	0.155	0.03	4.89	**<0.00001**
Development (with mites)	0.0002	0.005	0.047	0.96
Reproduction (with mites)	−0.007	0.005	−1.31	0.19
Brood size	**−0.002**	**0.0002**	**−11.25**	**<0.00001**
Carcass mass	**0.005**	**0.0013**	**4.49**	**<0.00001**
Female size	−0.006	0.005	−1.10	0.27
Experimental block	−0.005	0.003	1.53	0.12
Development (with mites) * Reproduction (with mites)	0.007	0.007	1.00	0.31

Explanatory variables	Coef	Exp(coef)	*SE*(coef)	*z*	*p*
*Female life span (Cox‐proportional hazards model with mixed effects)*					
Development (with mites)	0.28	1.33	0.33	0.85	0.39
Reproduction (with mites)	0.41	1.51	0.33	1.23	0.22
Carcass mass	−0.03	0.96	0.07	−0.44	0.66
Female size	−0.32	0.72	0.38	−0.85	0.39
Experimental block	−0.36	0.69	0.25	−1.43	0.15
Development (with mites) * Reproduction (with mites)	−0.25	0.77	0.45	−0.55	0.58

Full models are shown.

Bolded *p* values denote significant effect(s). Significance level is at 0.05.

**Figure 3:**
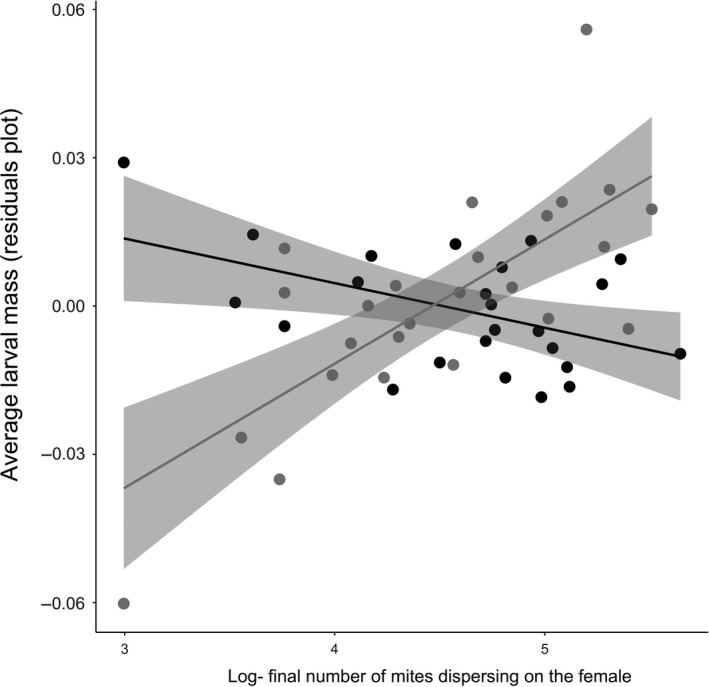
The relationship between the final number of second‐generation mites dispersing on the females (log‐transformed) and the average larval mass, when females developed with mites (gray data‐points) or without mites (black data‐points). Points show the partial residuals controlling for other explanatory factors in the analysis, with each data‐point representing the adult breeding event of one female. For this, we ran a general linear model including all the confounding variables (carcass mass, brood size, female size, and experimental block). After we plotted the residuals from this model (*y* axis) in relationship to the final number of second‐generation mites dispersing on the females in her adult breeding event (log‐transformed; *x* axis), separated by the female's developmental treatment (when females developed with mites (gray data‐points) or without mites (black data‐points)). The lines show the least squares regression between the two variables, separated by the developmental treatment (when females developed with mites (gray line) or without mites (black line)), and their respective 95% CI. Note that when considering only the mite presence/absence treatment, the average larval mass produced by the females was very similar (developing with mites, reproducing with mites mean = 0.138 g; median = 0.135; *SD* = 0.02; developing with mites, reproducing without mites mean = 0.14 g; median = 0.137; *SD* = 0.03; developing without mites, reproducing with mites mean = 0.137 g; median = 0.14; *SD* = 0.03; developing without mites, reproducing without mites mean = 0.137 g; median = 0.133; *SD* = 0.03)

We obtained different results when we analyzed the effect of the number of mites present at the end of reproduction (i.e., the number of second‐generation mites). This time, we found that females that developed alongside mites subsequently produced heavier larvae in their first breeding event when there was a higher density of second‐generation mites (Table 2; Figure 3). Note that this significant interaction was not caused by outliers: The interaction term remained significant when excluding the two extreme data‐points observed in Figure 3 (interaction effect: Estimate = 0.028, *SE* = 0.0059, *t = *4.80, *p* < 0.001). The greater the density of mites, the heavier the larvae they produced (effect of the log‐final density of second‐generation mites dispersing on the female on the average larval mass that she produced, for females that developed with mites and bred with mites: Estimate = 0.02, *SE* = 0.004, *t = *5.88, *p < *0.001). Females that did not develop alongside mites showed the opposite response. They produced slightly lighter larvae when mite density was higher (effect of the log‐final density of second‐generation mites dispersing on the female on the average larval mass that she produced, for females that developed with mites and bred with mites Estimate = −0.008, *SE* = 0.004, *t *= −2.05, *p = *0.054; after removing the outlier of 0.0637 g). We found no equivalent effects of mites on brood size (Table 2).

**Table 2:** Effect of mite density on female life‐history traits, considering only the females that bred alongside mites as adults

Explanatory variables	Estimate	*SE*	*t*	*p*
*Brood size (general linear model)*				
Intercept	2.50	25.12	0.10	0.92
Log‐final number of mites	−0.63	2.69	−0.23	0.81
Development (with mites)	−7.40	16.94	−0.43	0.66
Carcass mass	−0.18	0.83	−0.22	0.82
Experimental block	−3.58	2.51	−1.42	0.16
Female size	4.24	4.36	0.97	0.33
Development * log‐final number of mites	1.61	3.71	0.43	0.66

Explanatory variables	Estimate	*SE*	*t*	*p*
*Average larval mass (generalized least squares)*				
Intercept	**0.23**	**0.04**	**5.51**	**<0.0001**
Development (with mites)	**−0.16**	**0.02**	**−5.64**	**<0.0001**
Log‐final number of mites	**−0.01**	**0.004**	**−2.26**	**0.02**
Female size	−0.01	0.007	−1.59	0.11
Experimental block	−0.0002	0.004	−0.04	0.96
Brood size	**−0.002**	**0.0002**	**−10.29**	**<0.0001**
Carcass mass	**0.005**	**0.001**	**3.51**	**0.001**
Development (with mites) * log‐final number of mites	**0.03**	**0.006**	**5.74**	**<0.0001**

Explanatory variables	Coef	Exp(coef)	*SE*(coef)	*z*	*p*
*Female life span (Cox‐proportional hazards model)*					
Development (with mites)	0.14	1.15	0.34	0.45	0.67
Average larval mass (standardized)	−0.11	0.89	0.21	−0.53	0.59
Carcass mass	−0.04	0.95	0.11	−0.45	0.65
Experimental block	−0.30	0.73	0.33	−0.93	0.34
Female size	0.13	1.14	0.54	0.25	0.80
Development * average larval mass (standardized)	**0.75**	**2.12**	**0.33**	**2.23**	**0.02**

*n* = 25 females developing with mites and *n* = 25 females developing without mites. Full models are shown.

Bolded *p* values denote significant effect(s). Significance level is at 0.05.

### 3.2 Is the direction of the relationship between current reproductive investment and survival explained by exposure to mites during development?

**Figure 4:**
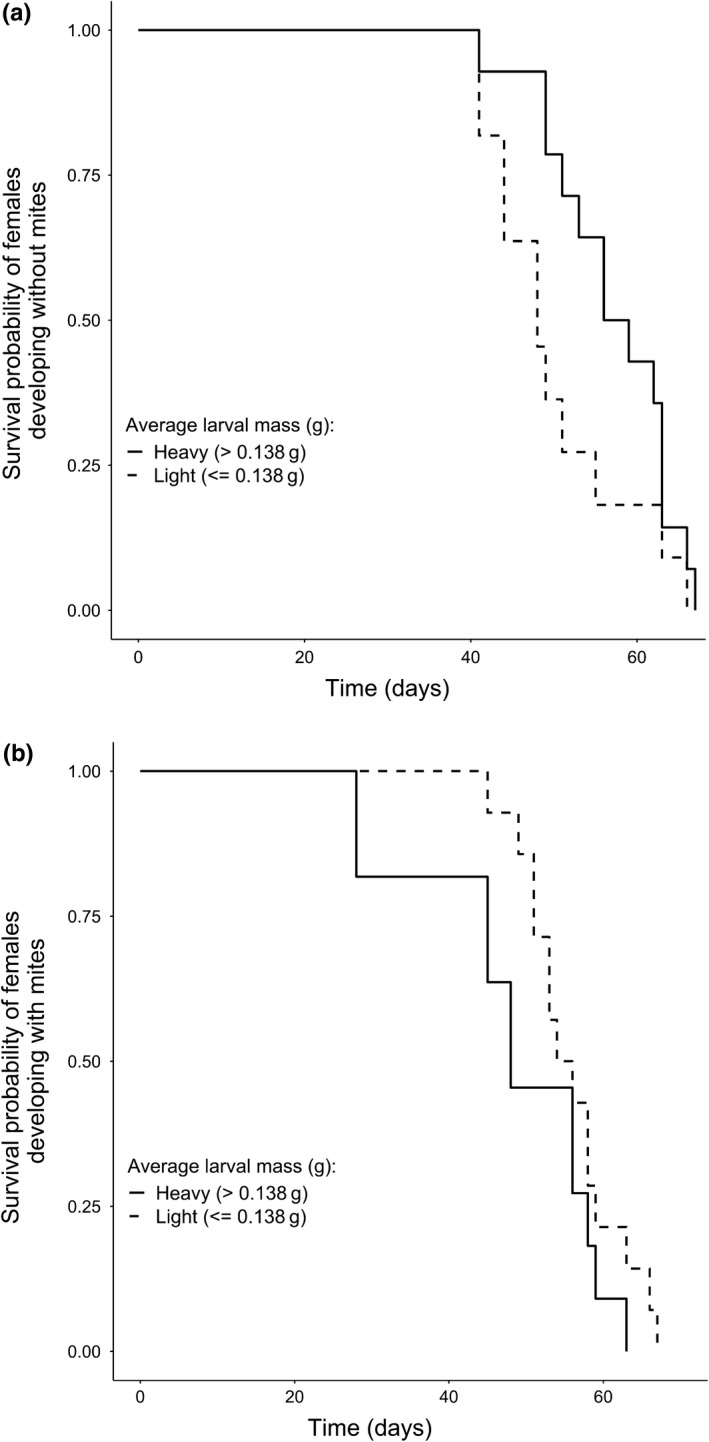
Survival curves for mothers producing heavy (continuous lines) or light (dotted lines) larvae, when females developed (a) without mites or (b) with mites. The dataset is separated by the median average larval mass (0.138 g)

The direction of the relationship between larval size and female survival differed according to whether or not females had been exposed to mites during development (considering only females that reproduced alongside mites as adults; Table 2; Figure 4). For females that did not develop alongside mites as larvae, those that produced heavier larvae had greater subsequent survival (Table 2; Figure 4). However, for females that developed in the presence of mites, the production of heavier larvae was associated with reduced subsequent survival. We checked whether this result was caused by outliers. However, the effect was qualitatively similar when we excluded females of particularly low quality (we had two females with a life span of <30 days, whereas all other females lived at least 40 days: Coef = 3.22; Exp(Coef) = 25.13; *SE*(Coef) = 1.48; *z* = 2.16; *p = *0.03). The interaction between the developmental condition of the females and the number of second‐generation mites (log‐transformed) did not influence the survival of the females (Coef = 0.43; Exp(Coef) = 1.55; *SE*(Coef) = 0.52; *z* = 0.83; *p = *0.40).

### 3.3 Does the early‐life environment change resource acquisition or provide important information in natural populations?

We found no difference in female size between those that developed alongside mites and those that did not in step one of the laboratory experiment (*χ* ^2^ = 1.30, *df *= 1, *p = *0.25). Nor could female size account for variation in the extent of current reproductive investment or female survival (Table 1).

**Figure 5:**
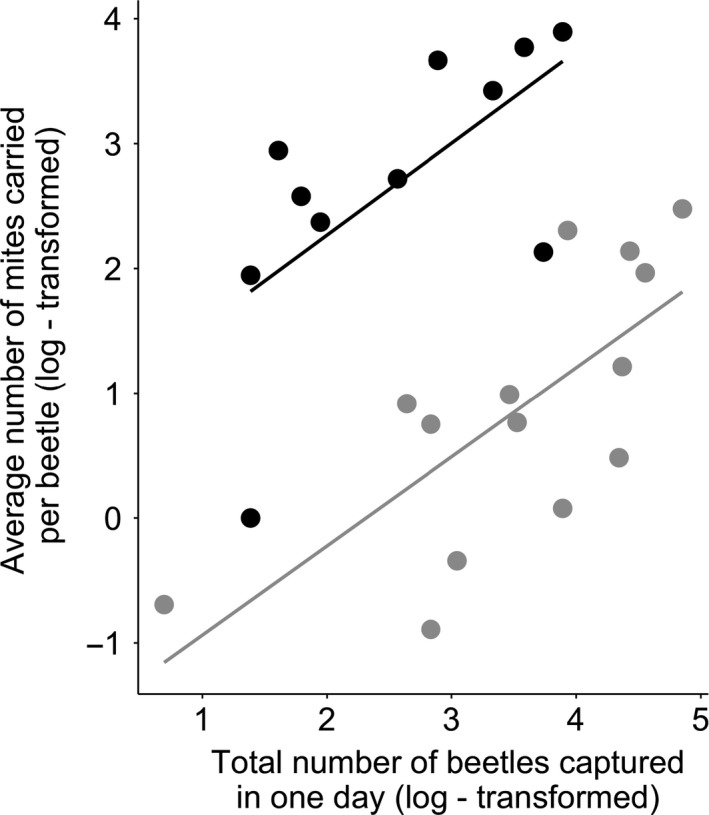
The relationship between the number of *Nicrophorus *spp *beetles* sampled in each field collection, and the average number of mites carried by each beetle. Each data‐point shows samples collected on a single day. The black points and black line show Byron's pool, and the gray points and gray line show Wicken Fen. The data have been log‐transformed. The lines show the least squares regression line between the two variables, by site

In the natural populations that we studied, we found a positive relationship between the average number of mites carried by each beetle on each collection date and the total number of beetles in the site, on that date (after removing the interaction from the model: Estimate = 0.72; *SE* = 0.16; *t *value = 4.38; *p = *0.0002; Figure 5). We did not find a significant interaction between the site and the total number of beetles collected in each date in each site (interaction effect: Estimate = −0.02; *SE* = 0.34; *t *value = −0.06; *p = *0.95). The intercepts of the sites were significantly different: there were many more mites per beetle in Byron's pool than in Wicken Fen (Effect of the site (Wicken Fen), after removing the interaction from the model: Estimate = −2.51; *SE* = 0.36; *t *value = −6.83; *p < *0.0001).

## 4 DISCUSSION

Our goal was to understand the role of the early‐life environment in explaining plasticity in a life‐history trait. We analyzed whether exposure to mites during early life and exposure to mites during first reproduction each independently influenced: (a) the extent of investment in current reproduction and (b) the direction of the relationship between current reproductive investment and survival after reproduction.

### 4.1 Plasticity in the extent of current reproductive investment

We found no evidence for our a priori expectation that the mere presence or absence of mites at either life stage would explain current levels of investment in reproduction (Table 1). Nor did we find any evidence that the presence or absence of mites could explain variation in beetle life span (Table 1). However, our post hoc analyses suggest that this is because beetles appear to base their life‐history decisions on the absolute number of mites present during their first bout of reproduction, rather than whether mites are simply present or absent. Although we added the same number of deutonymphs at the start of each “mite present” treatment, mite reproductive success was highly variable and the number of deutonymphs produced during the reproductive attempt varied by an order of magnitude (see Section 2). When we included this variation in mite reproductive success in our analyses, we found it could explain variation in average larval mass—our measure of investment in current reproduction by beetles (Figure 2, Table 2). However, the effect of mite number on average larval mass depended on the female's early‐life environment. If she had been reared alongside mites as a larva, the relationship was positive, but if she had not previously been exposed to mites, the relationship was negative (Figure 2). Therefore, we conclude that the early‐life environment changed the slope of the reaction norm relating mite number to average larval mass. More generally, our results suggest that the early‐life environment can explain the individual variation in life‐history plasticity that has been documented in natural populations of diverse species (Dingemanse & Wolf, 2013; Nussey et al., 2007).

### 4.2 Plasticity in the sign of the relationship between current reproductive investment and survival

In addition, we found that average mass of the larvae raised during first reproduction predicted the female's future survival (Table 2, Figure 4). However, this relationship differed depending on whether or not the female had been exposed to mites in her early life: It was only positive if a female developed without mites, but it was negative if she developed with mites (Figure 4). Therefore, we conclude that the early‐life environment also influences the direction of the relationship between investment in current reproduction and survival to breed again. Variation in the direction of this relationship is commonly seen within other species (e.g., Reznick et al., 2000), and our results suggest it might in part be attributable to variation in the early‐life environment.

### 4.3 The role of the early‐life environment: supplying resources or providing information?

We also considered two different ways in which exposure to mites in the early‐life environment could have influenced life‐history traits. We asked: Do mites impose constraints on females during their development, as suggested by somatic models; or are they a cue for beetle population density, as suggested by informational models (Frankenhuis & Panchanathan, 2011; Nettle & Bateson, 2015)? We are not aware of previous empirical work that has attempted to compare the merits of each type of model in explaining results from a single dataset. The key conclusion we draw from this exercise is that it is a difficult task for the empiricist. Perhaps in the real world, these functions are not mutually exclusive alternatives but instead are complementary processes for optimizing investment in current reproduction. To illustrate this general point, we set out below the specific interpretations of our data offered by each type of model.

To begin, let's assume that mites limit access to resources during development, as suggested by the somatic models. According to this view, females raised in a mite‐present environment might be doomed by their inferior quality to breed only once—especially if they encounter many mites again during their first bout of reproduction (if the negative effects of the mites increase with mite number). This would explain why these females put more effort into current reproduction (Figure 3) and survived less well thereafter (Figure 4). Conversely, better‐quality females, that developed without mites, might be more likely to breed twice. They can potentially withhold investment in current reproduction in a poor‐quality, mite‐rich environment in anticipation of being able to breed again (Figure 3). A key weakness with this interpretation is that we found no evidence that development alongside mites yields females of poor quality. It might be argued we should have used female fat or protein content to assess female quality, rather than female mass (Socha, 2006), and this remains to be done in future work.

The informational model offers a different interpretation. It suggests that mites provide information about the density of the burying beetle population, and therefore the likelihood that a beetle will have to fight for the key breeding resource (the carcass). Data we collected from natural populations are consistent with the idea that mites can act as a cue in the short‐term for local *Nicrophorus* population density (Figure 4). The greater the density of beetles, the greater the scale of competition for a carcass, and the less profitable it should be to withhold resources for an unlikely second breeding attempt. Double exposure to mites, during development and then again during first reproduction, increased the accuracy of this environmental information and made these females more likely to increase investment in current reproduction—potentially because they were more certain they would not breed again (Figure 3). Females that were exposed only once to mites had mixed information about environmental quality. They were more uncertain about their prospects for future reproduction; this caused them to revert to a default life‐history strategy based on their intrinsic quality, rather than unreliable extrinsic cues. Thus, in different ways, the informational and somatic models can each account for the results we found. We have no evidence so far that would allow us definitively to reject one model in favor of the other.

In summary, we have shown that the early‐life environment can adaptively account for individual variation in the extent of phenotypic plasticity in a key life‐history trait: investment in current reproduction. Previous analyses of individual variation in life‐history plasticity have mostly used long‐term datasets (Nussey et al., 2005; Przybylo, Sheldon, & Merilä, 2000). Our experimental results complement this work by demonstrating a causal influence of the early‐life environment on individual variation in life‐history plasticity. In addition, we have shown that the early‐life environment determines whether different aspects of the adult phenotype trade‐off with one another, or are positively correlated. However, whether the early‐life environment influences the extent of plasticity because it supplies key resources, or key information, or both, is very difficult to determine empirically. Nevertheless, in general, our findings suggest there are nuanced ways in which the early‐life environment can induce phenotypic variation in adults, which deserve more attention in future work.

## CONFLICT OF INTERESTS

None declared.

## AUTHORS CONTRIBUTIONS

ODG designed the laboratory experiment. ODG and AD executed the laboratory experiment. ODG and AA designed and executed the field experiment. ODG analyzed the data. ODG, AD, AA, SE, and RMK discussed the results interpretation and wrote the paper.

## DATA ACCESSIBILITY

The data supporting this paper can be found in Dryad https://doi.org/10.5061/dryad.v57471h.
